# Navigating the Paradox of Creativity: Pathways to Fostering Talent and Innovation

**DOI:** 10.3390/bs16010129

**Published:** 2026-01-16

**Authors:** Lin Huang, Yan Sun, Chenchen Zhang, Yong Shao, Yuan Yuan, Wangbing Shen

**Affiliations:** 1 School of Music and Dance, Nanjing Normal University of Special Education, Nanjing 210038, China; 2 School of Education Science, Nanjing Normal University, Nanjing 210024, China; 3 School of Literature, Nanjing Normal University, Nanjing 210024, China; 4 Academy of Jiangnan Culture, Jiangnan University, Wuxi 214100, China; 5 School of Government, Nanjing University, Nanjing 210023, China; 6 School of Special Education, Nanjing Normal University of Special Education, Nanjing 210038, China; 7 School of Public Administration, Hohai University, Nanjing 211100, China

**Keywords:** creativity, dual-pathway model, flexibility, usefulness, hyperscanning

## Abstract

Creativity serves as a fundamental driver of human learning, personal development, and societal progress. This study synthesizes recent empirical and theoretical advances in educational psychology and creativity neuroscience to characterize the paradoxical nature of creative processes. We conceptualize creativity through three interdependent dimensions—novelty with usefulness, persistence alongside flexibility, and divergence in convergence—illuminating both its cognitive architecture and neurophysiological dynamics. By integrating evidence across levels, we bridge individual cognitive mechanisms with group dynamics and cultural contexts to propose actionable strategies for cultivating creativity. These findings offer critical insights into how these dimensions operate synergistically, informing the design of educational and applied interventions that promote sustained, adaptive creative development.

## 1. Introduction

What if the very conditions that spark creativity are also the forces that stifle it? This paradoxical question captures one of the most perplexing phenomena in psychology. For decades, researchers have celebrated creativity as a hallmark trait of human wisdom—the wellspring of technological innovation, scientific discovery, and cultural transformation (Wiggins & Bhattacharya, 2014). Yet deeper inquiry reveals a disturbing pattern: the very forces that catalyze creative breakthroughs also constrain them. People champion creativity while actively rejecting innovative ideas (Mueller et al., 2012). Organizations establish innovation mechanisms that inadvertently suppress novelty (Amabile et al., 1996). Teachers profess support for creative students while autonomously discouraging creative behaviors (Westby & Dawson, 1995). These contradictions point to something fundamental: creativity possesses an inherent paradox, composed of opposing forces that must coexist in dynamic tension rather than reconcile into stable equilibrium.

Recognizing creativity as inherently paradoxical marks a conceptual shift. Traditional theories have often depicted creativity as the synthesis or integration of disparate ideas—a harmonious combination of previously unconnected elements. Such metaphors presuppose compatibility. Paradox, in contrast, embraces the juxtaposition of seemingly irreconcilable conflicts (Brillenburg Wurth, 2019; Smith & Lewis, 2011). This distinction proves crucial: if creativity involves paradox rather than simple combination, then resolving tension by choosing one extreme over another—novelty versus usefulness, flexibility versus persistence, divergence versus convergence—fundamentally misunderstands its essence. The paradox is not a defect to be corrected but the very ground from which creative thought arises. Empirical evidence underscores this view. The well-known “mad genius paradox” illustrates how the most creative individuals exhibit higher risks of psychopathology (Simonton, 2014; Zhao et al., 2022). Recent findings further indicate that highly creative persons dynamically oscillate between opposing cognitive and affective states—order and chaos, persistence and flexibility—rather than seeking balance at a midpoint (Lambert, 2020; Zabelina & Robinson, 2010). This pattern mirrors the complexity of adaptive systems, implying that creativity may be an emergent property of paradoxical dynamics.

At the ontological level, creativity is defined by interdependent opposites that cannot exist in isolation. Consider the enduring tension between novelty and usefulness: both are essential, yet they inherently conflict. Radical novelty can undermine practical value, while excessive utility constrains originality within established norms (Simonton, 2012; Shen et al., 2019; Harvey & Berry, 2023). The model of creativity proposed by Simonton (2012) captures this interdependence, contending that creativity is not a sum of novelty and usefulness but their product, further modulated by surprise. Because each dimension amplifies or attenuates the others, optimizing creativity requires simultaneous—not sequential—coordination of conflicting goals. This explains why creativity is so rare and demands such intense cognitive effort: it requires sustaining productive disequilibrium. Practically, paradoxes carry dual consequences, demanding sophisticated navigation. Although the paradoxical framework can enhance creativity by stimulating exploration of novel and practical solutions, it may also trigger anxiety, defensiveness, and clinging to old patterns (Calic & Hélie, 2018). Computational models of creative cognition indeed reveal that balanced paradoxical tension enhances originality and novelty, while imbalanced states—whether characterized by excessive or insufficient conflict—impair cognitive output. Individuals with high dispositional creativity may even experience diminishing returns under unmoderated tension, as the proliferation of alternatives delays insight and resolution (Calic & Hélie, 2018; Bonetto et al., 2021). Thus, the paradox requires not resolution but mastery—a dynamic capability that treats both extremes as complementary sources of creative energy.

Understanding creativity as inherently paradoxical holds profound implications for both research and practice. This necessitates moving beyond dichotomous mindset that views opposing forces as mutually exclusive choices, toward a mindset that embraces contradiction—regarding contradictions as creative tensions that stimulate innovative vitality (Kearney et al., 2019; Andriopoulos & Lewis, 2010). Paradox theory provides the theoretical framework for this shift, emphasizing that conflicting demands possess both competitive and complementary qualities. The “paradoxical mindset” captures this cognitive-emotional orientation: the ability to embrace tension as an energy source rather than resist it as a threat (Miron-Spektor et al., 2018; Liu et al., 2020). Recent studies show that such a mindset fosters innovation, learning, and professional growth, particularly when reinforced by leaders who model paradoxical thinking themselves (e.g., Liu et al., 2020). As a result, paradox is not obstacles to creativity but a key foundation—the condition making creativity possible.

Despite growing recognition of these dynamics, systematic reviews of the paradox of creativity remain scarce. Existing syntheses have largely focused on specific phenomena, such as the “mad genius” paradox (e.g., Brüne, 2004; Galang, 2010; Agnati et al., 2012; Thys et al., 2013) or organizational ambidexterity (Calic et al., 2020; Manca, 2022; Volpentesta et al., 2023), without addressing the ontological paradoxes underlying the creative process itself. The paradox of creativity refers to constitutive contradictions inherent in creative phenomena: tensions arising from simultaneously necessary yet mutually constraining requirements (Guyon et al., 2018). A genuine paradox meets three conditions: (1) both elements are simultaneously necessary, (2) each restricts the cognitive and operational space of the other, creating structural tension at conceptual, perceptual, procedural, or neurophysiological levels, and (3) success requires working within—not resolving—this constraint structure. This differs fundamentally from Janusian integration and ambidexterity. Janusian thinking synthesizes opposites into coherent wholes that resolve opposition (Rothenberg, 1996); paradoxes maintain irreducible tension. Ambidexterity develops separate capabilities deployed contextually (Revilla & Rodriguez-Prado, 2018); paradoxes demand simultaneous activation of competing constraints. As mentioned above, creative products must be both novel and useful, yet pursuing novelty undermines usefulness (deviation from established utility) while pursuing usefulness undermines novelty (adherence to proven patterns). Theoretically, Knoblich and Haider’s (2019) constraint relaxation theory illuminates this at both conceptual and perceptual levels. In the nine-dot problem, perceptual constraints (lines within boundaries) and conceptual constraints (connecting all dots) are co-active restrictions where relaxing one is necessary but insufficient—the solver must satisfy both despite their incompatibility. The solver cannot simply “balance” these constraints or find a middle ground; they must discover the specific configuration where both constraints are satisfied despite their apparent incompatibility. Neurophysiologically, this manifests in the antagonistic activation patterns between the default mode network (supporting novelty generation) and executive control network (supporting usefulness). These networks show mutually inhibitory connectivity—activating one suppresses the other—yet creative insights require their coordinated activation, creating genuine paradox.

Current research has insufficiently integrated these perspectives to address tensions within creative processes. A critical gap remains: how do contradictory demands coexist and interact within creative process? This study synthesizes advances in educational psychology and creativity neuroscience to characterize the paradoxical nature of creative processes, examining: (1) What core paradoxical dimensions define creative processes? (2) How do cognitive and neurophysiological mechanisms support these paradoxes? Integrating conceptual and bibliometric analyses, we examine three interrelated paradoxes: novel and useful—ideas must be both original and contextually useful; persistent yet flexible—creative work demands sustained effort alongside openness to change; and divergent yet resonant—creative work must balance individual distinctiveness with collective coherence. These paradoxes are not separate categories but interwoven forces within complex adaptive systems, shaping creative cognition, collaboration, and innovation ecosystems. The challenge is not eliminating these tensions but developing capacity for constructive engagement with them.

To achieve these aims, the study unfolds in three stages. We begin with a bibliometric analysis of the paradox of creativity using the Web of Science corpus (retrieved October 2025), tracing its intellectual evolution and disciplinary diffusion. We then undertake a narrative synthesis of major empirical and theoretical contributions that have shaped contemporary discourse. Building on these foundations, the paper explores three emerging but critical paradoxes in depth and extracts their implications for creative education, innovation training, and the cultivation of creativity. Through this inquiry, we advance the field as follows. First, we offer a systematic theoretical integration of different paradoxes of creativity, moving beyond epistemological or practical perspectives to reveal the constitutive contradictions embedded in the very nature of creativity phenomena. Second, we synthesize multilevel evidence from psychological and neurocognitive mechanisms to social and collaborative dynamics, revealing how novelty–usefulness, persistence–flexibility, and diversity–resonance paradoxes function as interdependent forces sustaining creativity emergence. Third, we translate these insights into actionable educational principles, illustrating how paradox-informed pedagogies nurture creativity by embracing rather than resolving fundamental tensions. By integrating evidence across levels and bridging theory with practice, this study advances understanding of creativity as an emergent property of paradoxical systems and charts empirically grounded pathways for 21st-century creativity education.

## 2. Method

The study is twofold in methodology. We first used bibliometric analysis to scan and scope existing studies to explore the potential issues on the paradoxes of creativity and then conduct a narrative analysis to investigate the paradoxes of creativity in details. Bibliometrics provides a quantitative framework for mapping scholarly output, intellectual structures, and emerging trends within research domains (Ma & Ismail, 2025). Narrative analysis, by contrast, takes a qualitative approach to understanding how meaning is constructed through storytelling across various text forms—from personal accounts and fictional works to historical records and organizational narratives (Baumeister & Leary, 1997).

This investigation draws on bibliometric data sourced from the Web of Science Core Collection in October 2025. As a well-known and gold-standard repository for scholarly literature and citation networks, Web of Science serves as the cornerstone database for bibliometric investigations across disciplines, covering most mainstream and influential studies from a diversity of journals indexed by Social Sciences Citation Index (SSCI) or Science Citation Index Expanded (SCI-E), which also exhibited strengths in coverage quality and analytical reliability (Ma & Ismail, 2025; C. Zhang et al., 2025). We conducted a comprehensive search within the Web of Science Core Collection, specifically targeting the SSCI (2004–present) and SCI-E (1996–present). To descriptively capture how the creativity research community itself conceptualizes and labels paradoxical phenomena through peer-validated publications—a legitimate research question distinct from prescriptively adhering to particular philosophical definitions—we deliberately designed the most transparent and replicable inclusion criterion. Specifically, we included only studies that explicitly and directly employ “paradox” as their framework, while systematically excluding implicit terms such as “tension” or “duality” that would necessitate subjective interpretation. This methodological choice ensures our analysis reflects the field’s own scholarly discourse as manifested through its formal publication practices. Using the “All Fields” search parameter, we performed a combined query for *paradox* AND *creativity*. In terms of analysis, we adopted a dual-pronged analytical strategy: performance analysis to quantify scholarly contributions and gauge research impact (Donthu et al., 2021), coupled with science mapping to trace the intellectual architecture and evolutionary trajectories shaping the field (Heradio et al., 2016).

## 3. Results

### 3.1. Sources Used and Their Structure

We queried the Web of Science Core Collection (SSCI 2004-present; SCI-E 1996-present) using paradox AND creativity across all fields, retrieving 335 records: 306 articles, 18 reviews, and 5 book reviews. The article subset included 5 conference proceedings, 1 retracted publication, and 6 editorials. Most papers were in English (*n* = 322), with 13 in other languages: Afrikaans (*n* = 1), French (*n* = 4), German (*n* = 3), Italian (*n* = 1), Lithuanian (*n* = 1), Russian (*n* = 1), and Spanish (*N* = 2). After removing the retracted paper and book reviews, we retained 329 records spanning 1998-2025 (no publications in 1999 or 2001). These appeared in 200 sources, with 21 journals publishing multiple articles (Table 1), and distributed across 77 research domains (Table 2 shows areas with *n* ≥ 3), indicating a moderate concentration of scholarship within specific venues.

The publication analysis indicates that research on the creativity paradox is primarily concentrated in journals focusing on creativity, psychology, and organizational behavior. The most frequently cited source is *Creativity and Innovation Management* (*n* = 19), followed by *Frontiers in Psychology* (*n* = 12), *Journal of Creative Behavior* (*N* = 11), and *Creativity Research Journal* (*n* = 9). Organizational and management perspectives are also prominent, with journals such as *Organizational Behavior and Human Decision Processes* (*n* = 9), *Journal of Organizational Behavior* (*n* = 6), *Human Relations* (*n* = 5), *Journal of Product Innovation Management* (*n* = 4), and *Organization Studies* (*n* = 4) contributing substantially. Additionally, interdisciplinary outlets—including *Psychology of Aesthetics, Creativity, and the Arts* (*n* = 7), *Current Psychology* (*n* = 4), and *Neuroscience and Biobehavioral Reviews* (*n* = 3)—illustrate growing integration between psychological, artistic, and neuroscientific approaches. Further, Table 2 shows that these studies are predominantly situated within the fields of Business & Economics and Psychology. *Business & Economics* is the most frequently represented area (*n* = 101) while *Psychology* follows (*n* = 58). Several interdisciplinary overlaps—such as *Psychology; Business & Economics* (*n* = 13), *Business & Economics; Social Sciences–Other Topics* (*n* = 10), and *Business & Economics; Engineering* (*n* = 8). Secondary clusters appear in *Education & Educational Research* (*n* = 10), *Neurosciences & Neurology* (*n* = 5), and *Cultural Studies* (*n* = 4), suggesting broader applications in learning, brain research, and cultural contexts.

**Figure 1:**
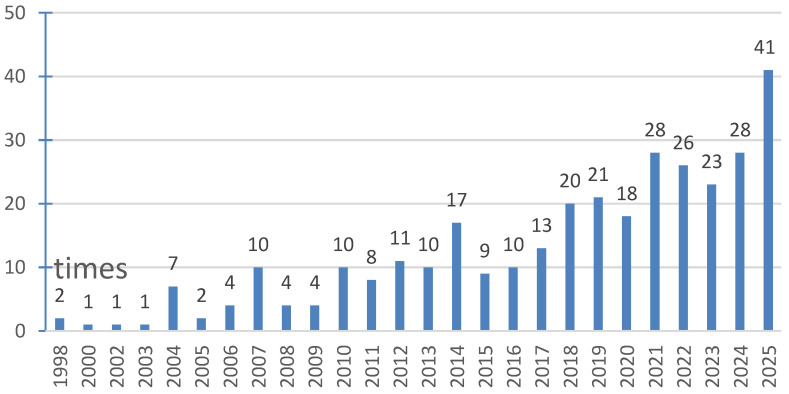
Yearly distribution of the analyzed articles.

Figure 1 illustrates the temporal evolution of research addressing the creativity paradox. The earliest influential work emerged in 1998, followed by sporadic publications through 2005. A gradual upward trajectory became evident starting in 2006, with sustained growth (in absolute account) materializing after 2010. The field has experienced particularly pronounced expansion since 2018, consistently generating over 20 publications annually, with research output reaching 41 papers in 2025. However, this growth pattern warrants careful interpretation. The observed increase in paradox-focused publications may reflect several converging trends rather than a singular phenomenon. First, creativity research has undergone substantial expansion during this period, potentially inflating absolute publication counts across all subdomains. Second, organizational and business scholars have demonstrated heightened interest in paradox frameworks more broadly, which may have amplified paradox-related publications independent of creativity-specific developments. Consequently, while the absolute number of creativity paradox studies has risen, this trend should be contextualized within the broader proliferation of both creativity scholarship and paradox-oriented research methodologies. The acceleration since 2018 nevertheless suggests that the creativity paradox has emerged as a substantive focal point within the field, meriting systematic examination beyond what might be attributed solely to general disciplinary growth.

To ensure topical centrality, we isolated studies featuring paradox in titles (*n* = 122) or abstracts (*n* = 234). After excluding 77 peripheral studies (a paradox was not mentioned in titles or abstracts), 6 removed records above, 3 unrelated reviews, and 1 misclassified qualitative study, a final sample of 249 studies was used for narrative analysis. The analysis on the paradoxes of creativity showed that creativity inherently involves tensions and contradictions that are not obstacles to overcome but dynamic forces to navigate. Rather than emerging despite paradoxes, creativity is realized through them. Also, the paradox is multidimensional, typically manifested in creativity as a structure of complementarity between novelty and utility, persistence and flexibility, individual diversity and group resonance, namely three critical paradoxes mentioned above—that together defines the very essence of creativity and delimits its practical realization (see Table 3). Additionally, these studies converge on the view that paradox is the ontological essence of creativity, shaping its processes, outputs, and outcomes across individual, team, and organizational levels. The way individuals, teams, and organizations manage such tensions is influenced by moderators and boundary conditions: characters, expertise, and cultural background at the individual level; interdependence, and size at the team level (Osorio & Bornmann, 2021); and organizational factors such as climate, resources, and leadership. When effectively managed, paradoxes foster creativity, innovation, and adaptive performance, but when mishandled, they induce anxiety, conflict, and defensiveness—often displaying nonlinear, inverted U-shaped effects (Geng et al., 2025; Calic et al., 2019).

### 3.2. Three Important Paradoxes of Creativity

To derive deeper insights for creativity education, innovative talent development, and the cultivation of creative thinking, this section integrates prior research and bibliometric evidence to conduct an in-depth analysis of three core paradoxes of creativity. By unpacking these fundamental tensions that define and drive the creative process, the discussion would clearly reveal how embracing such paradoxes can unlock richer, more sustainable forms of creative growth.

#### 3.2.1. The Novelty–Usefulness Paradox

The novelty–usefulness paradox constitutes the ontological core of creativity, manifesting as an irreconcilable tension between two definitional requirements that must coexist yet are inherently in conflict. Creativity receives its most standard and established definition through conjoint criteria: outcomes must be both novel—breaking from convention—and useful—meeting practical needs or fulfilling meaningful functions (Runco & Jaeger, 2012; Simonton, 2012). This dual mandate frames creativity as adaptive originality wherein innovation generates demonstrable value (Weisberg, 2015). Yet herein lies the paradox: maximizing novelty often reduces usefulness by generating ideas too radical for implementation, while maximizing usefulness constrains novelty by anchoring thinking to existing frameworks. Simonton adds “surprise” as the third essential element in his three-factor definition, specifying that creativity based on novelty, usefulness, and surprise must be quantitative and multiplicative rather than additive (Simonton, 2012). This multiplicative specification is crucial: excelling on one dimension while failing on another yields near-zero overall creativity. A perfectly novel but useless idea contributes nothing; a useful but entirely conventional solution similarly fails to qualify as creative. This multiplicative relationship explains why genuine creative achievement remains so difficult—it demands simultaneous optimization of competing objectives rather than sequential addressing of separate requirements.

Integrating novelty and usefulness reveals a fundamental neural paradox: brain regions supporting novelty generation (default mode network, DMN) and usefulness evaluation (executive control network, ECN) exhibit antagonistic activation patterns—activating one suppresses the other. This antagonism manifests in the differential deactivation profiles of the DMN: familiar and useful stimuli produce attenuated suppression, enabling spontaneous associative processing, while novel or useless stimuli elicit pronounced deactivation as cognitive control demands increase (Huang et al., 2015, 2018). Yet creativity require coordinated activation of these mutually inhibitory networks. The temporoparietal junction serves as a convergence hub for contextual updating and reorienting attention toward alternatives, while the anterior cingulate cortex and left prefrontal cortex monitor and resolve representational conflicts between established and novel schemas, particularly when novelty and usefulness co-occur (Huang et al., 2018). The middle temporal gyri-hippocampus pathway further facilitates novel association formation by dissolving conceptual boundaries (Ren et al., 2020). This paradoxical coordination—wherein ECN and DMN modulation reflects dynamic balance between spontaneous generation and cognitive control (Shen et al., 2017; Dietrich & Kanso, 2010)—underscores that creativity arises not from resolving but from sustaining this tension: novelty generates fresh perspectives while usefulness grounds them in relevance, each dimension simultaneously enabling and constraining the other. Ideas that are novel but not useful remain eccentric; those useful but not novel prove routine. High-impact creativity produces “difference that makes a difference,” reshaping problems, evaluative standards, and entire domains. The multiplicative relationship between dimensions means that strategies optimizing one while sacrificing the other prove fundamentally misguided. Educational and organizational contexts must cultivate “paradox competence”—capacity to sustain cognitive and affective engagement with contradictory demands without premature resolution toward either pole. Rather than viewing usefulness constraints as creativity inhibitors or novelty generation as divorced from practical consideration, productive approaches recognize their dynamic interdependence: usefulness provides grounding enabling sustained novelty exploration, while novelty infuses usefulness with transformative potential. The paradox persists not as problem to solve but as creative tension to leverage—the generative friction from which genuine innovation emerges.

#### 3.2.2. The Persistence–Flexibility Paradox

The persistence–flexibility paradox captures a fundamental temporal and regulatory tension in creative work: creativity demands exploratory flexibility—the capacity to generate multiple possibilities, shift perspectives, and embrace ambiguity—while simultaneously requiring exploitative persistence—sustained focus, iterative refinement, and tolerance for frustration inherent in developing initial insights into robust solutions (Steele et al., 2021). This dual requirement reflects that creative accomplishment emerges from sustained effort guided by strategic adaptability. As an example, Thomas Edison’s iterative development of the electric light bulb, involving over a thousand experiments, exemplifies how breakthroughs arise from relentless experimentation rather than immediate insights. Yet without flexibility, persistence risks devolving into counterproductive rigidity. Creative work unfolds in uncertain contexts where solutions remain ambiguous and initially misrepresented, requiring cognitive and behavioral flexibility to transcend mental fixation through restructuring and pursue alternative trajectories when established strategies fail (Fink & Benedek, 2014). The paradox deepens temporally: creative processes cycle between divergent phases requiring flexibility and convergent phases requiring persistence, yet these phases cannot be cleanly separated (Reif et al., 2025). Premature convergence stifles possibilities while excessive divergence prevents coherent outcomes, creating “transit” challenges in moving from ideation to implementation.

Research on goal orientations reveals how motivational frames shape navigation of this tension (see Table 3). Exploration effort positively relates to product novelty while exploitation effort positively relates to product usefulness, with mastery-approach goal orientation promoting both types of effort but performance-approach orientation decreasing exploration effort (Steele et al., 2021). Individuals oriented toward mastery—developing competence and understanding—sustain both exploratory and exploitative effort because both contribute to learning, whereas individuals oriented toward performance—demonstrating ability relative to others—avoid exploration’s risks, preferring exploitation of known strengths. Studies of temporal leadership illustrate contextual complexity, revealing inverted U-shaped relationships particularly for teams with high knowledge complexity (Duan et al., 2023). Moderate temporal leadership—establishing pacing while allowing flexibility—optimizes creativity, whereas very low temporal leadership provides insufficient structure, leaving teams adrift, and very high temporal leadership over-constrains, suppressing exploratory flexibility necessary for breakthroughs. Research on ambidextrous leadership demonstrates that leaders simultaneously enacting seemingly opposing behaviors—being directive yet empowering, setting clear direction while encouraging experimentation—generate higher innovation than leaders emphasizing either pole (Wang et al., 2024; Kamil et al., 2025). This finding connects to paradox theory that paradoxical tensions become more salient, and paradoxical management more critical, as task complexity increases (Potonik et al., 2022): simple tasks may succeed through linear approaches emphasizing either flexibility or persistence, but complex activities demand dynamic oscillation between both poles.

Neuropsychologically, the persistence–flexibility interplay manifests through dynamic interaction between the DMN and ECN. The DMN supports associative, spontaneous ideation, whereas the ECN underlies top-down control, goal maintenance, and evaluative regulation (Beaty et al., 2015, 2016). Flexibility, marked by frequent shifts in attention or idea space, engages the DMN and posterior parietal cortex, facilitating spontaneous associative processing; persistence, characterized by sustained focus on a single idea or representational chunk, recruits the ECN—particularly dorsolateral prefrontal cortex and inferior frontal gyrus—supporting goal-directed memory search and cognitive inhibition (W. Zhang et al., 2020). Dual-task paradigms demonstrate that both shift frequency (flexibility) and dwell time (persistence) independently predict creative output (Wu & Koutstaal, 2022), emphasizing that dynamic interplay—not dominance of either mode—proves critical. Converging evidence indicates that creativity emerges from cross-network integration between DMN and ECN (Beaty et al., 2019; W. Zhang et al., 2020), allowing exploratory ideation to be guided by metacognitive control. Task context further shapes this balance: divergent thinking benefits from alternating flexibility and persistence, whereas convergent thinking relies more heavily on sustained persistence.

The Dual Pathway to Creativity Model (Nijstad et al., 2010) formally specifies this interplay, delineating two distinct yet interacting routes toward creative production. The persistence pathway involves systematic, effortful processing and sustained task engagement; the flexibility pathway mobilizes broad associative thinking and representational restructuring. While either pathway can independently generate creative outcomes, their integration yields more robust and context-responsive solutions. Creators sustaining deep engagement while flexibly adjusting strategies prove optimally equipped to navigate complex, ill-defined problems. In collaborative contexts, this dual engagement becomes especially consequential: persistence preserves collective focus and coherence; flexibility enables integration of heterogeneous perspectives, conflict resolution, and strategic recalibration. High-impact creators exhibit neither pure persistence nor mere open-mindedness; they discern when to intensify effort and when to recalibrate direction. Across scientific, artistic, and educational domains, achievement depends on enduring without rigidity and adapting without losing coherence. The paradox persists not as problem requiring resolution but as dynamic balance demanding continuous calibration. Optimal creativity emerges not from favoring one pole but from fluid oscillation responsive to contextual requirements, manifesting both behaviorally and physiologically in how ideas are generated, refined, and actualized.

#### 3.2.3. The Diversity–Resonance Paradox

Group creativity presents a fundamental ontological paradox: it cannot be reduced to the simple aggregation of individual contributions, yet it requires cognitive diversity to transcend individual limitations. This paradox challenges conventional assumptions that diversity per se drives creative outcomes. Creativity emerges from the dynamic integration of cognitive variability through shared understanding and coordinated engagement—what we term the diversity–resonance paradox. Divergence supplies novel inputs by expanding possibility spaces, while resonance integrates and transforms these inputs into coherent, actionable innovations. Creative collaboration thus represents not the multiplication of ideas but their orchestration, an emergent property enabled through the alignment of cognitive differences.

Cognitive and experiential heterogeneity—encompassing varied knowledge bases, cultural backgrounds, and problem-solving heuristics—expands solution spaces and enriches ideational pools (O’Reilly et al., 1998; Joshi & Roh, 2009). Such diversity enhances ideational fluency (Michinov, 2012), stimulates constructive conflict (Jehn & Mannix, 2001), and sustains innovation (West & Richter, 2011). Yet divergence alone determines neither creative success nor failure. Without mechanisms for alignment, diversity generates interpersonal friction, conceptual fragmentation, and cognitive overload. Research on paradoxical frames reveals this constraint: teams adopting paradoxical mental templates—frameworks recognizing and embracing contradictions—combined with high epistemic motivation develop more creative solutions than teams with paradoxical frames but low epistemic motivation (Miron-Spektor et al., 2022). The mechanism is that epistemically motivated teams with paradoxical frames actively exchange, consider, and integrate diverse ideas rather than settling for superficial compromises (Miron-Spektor et al., 2022). Studies of multicultural teams extend these insights, showing that members holding high multicultural paradox mindsets—accepting of and energized by intercultural tensions—exhibit enhanced creativity through information elaboration (Mannucci & Shalley, 2022; Shen & Yuan, 2015). Cultures valuing harmony may inadvertently suppress productive engagement with divergent perspectives, encouraging premature convergence (Leung et al., 2018; Geng et al., 2025). The challenge involves sustaining creative tension through alternating consensus learning—incorporating others’ ideas—and conflict learning—asserting one’s own ideas—thereby reinforcing norms of both collaboration and autonomy (Schoug et al., 2025).

Resonance reflects the emergent coordination of goals, emotions, and intentions among collaborators, scaffolded by shared mental models, interpersonal trust, and psychological safety. Critically, resonance manifests at the neural level. Hyperscanning studies reveal that inter-brain neural synchrony (INS)—real-time alignment of brain activity across individuals—correlates with joint attention, shared intentionality, and co-regulated problem-solving (Lu et al., 2023; Y. Zhang et al., 2024). This neural alignment supports predictive processing, mentalizing, and adaptive coordination, enabling groups to navigate complexity without suppressing individual contributions. Many studies empirically demonstrate that cognitive divergence enhances group creativity only when fostering interpersonal resonance. For example, Lu et al. (2023) stratified dyads by divergent creativity profiles performing a product poster task and found that Low–Low dyads matched High–High pairs in creative performance, driven by stronger INS in right dorsolateral prefrontal cortex and right temporoparietal junction—regions critical for joint attention and theory of mind. These dyads exhibited richer behavioral coordination, suggesting evenly matched capacities strengthen mutual adaptation. Further, Lu et al. (2021) found that moderately diverse educational teams achieved optimal flexibility and highest INS in left frontopolar area, whereas extremely homogeneous or diverse teams proved less effective. Optimal divergence—neither minimal nor maximal—promotes alignment of neural and creative processes, enabling groups to translate variability into coherent innovations.

Recent studies examining three-person teams during co-creation tasks reveal that high-creative teams exhibit stronger, bidirectional INS networks in bilateral dorsolateral prefrontal cortex and frontoparietal hubs, reflecting fluid integration of diverse perspectives (e.g., Lu et al., 2023). These neural patterns align with task phases such as idea elaboration and evaluation, demonstrating temporal and functional coordination. A consistent pattern emerges across studies: (educational) divergence amplifies creativity exclusively when supporting shared mindsets and dynamic neural alignment, particularly high diversity benefits cognitive flexibility (e.g., Lu et al., 2021). Without this alignment, divergence becomes noise rather than asset. Empirical evidence consistently links higher INS to more original, useful, and coherent outcomes (Lu et al., 2021, 2023), highlighting that divergence alone is insufficient—without resonance, diverse ideas remain unconnected, producing fragmentation rather than innovation. Practices such as co-sketching, storytelling, and neurofeedback platforms can amplify alignment by fostering engagement, emotional attunement, and coordinated problem-solving. Optimizing collective creativity thus requires intentionally designed environments supporting both diversity and dynamic alignment. The creative power of teams lies not in mere heterogeneity but in the skillful orchestration of difference through psychological and neural resonance, where creative collaboration operates less through compromise than through harmonization of divergent perspectives into shared cognitive rhythm.

## 4. Conclusions and Implications

Creativity emerges not as a unitary trait but as dynamic orchestration of paradoxical tensions operating across multiple dimensions. The novelty–usefulness paradox establishes the definitional core of creativity: outcomes must simultaneously break from convention and address meaningful needs, with their multiplicative relationship demanding concurrent optimization rather than sequential compromise (Runco & Jaeger, 2012; Simonton, 2012). The persistence–flexibility paradox captures temporal and regulatory dynamics: creative accomplishment demands both exploratory flexibility to pioneer possibilities and exploitative persistence to refine insights into robust solutions, manifesting physiologically through coordinated interaction between the DMN and the ECN (Beaty et al., 2015; Nijstad et al., 2010). The diversity–resonance paradox reveals social and collaborative dimensions: cognitive heterogeneity expands solution spaces, yet divergence amplifies creativity only when teams achieve neural and psychological resonance, integrating varied perspectives through shared mental models and inter-brain synchrony (Lu et al., 2021, 2023). These paradoxes are not problems requiring resolution but constitutive tensions demanding continuous calibration wherein genuine innovation emerges.

The novelty–usefulness paradox demands that educational contexts cultivate “paradox competence”—the capacity to sustain engagement with contradictory demands without premature resolution toward either pole. Instructional architectures must simultaneously engage executive and reward-related systems by presenting unfamiliar challenges demanding restructuring while reinforcing problem-solving through meaningful feedback, thereby amplifying both novelty and usefulness (Huang et al., 2018; Shen et al., 2016). This requires architecting constraint-driven tasks where learners address challenges using minimal resources or express layered concepts through simple forms—analogies, metaphors, prototypes—engaging metacognitive monitoring while pursuing elegant solutions (Tromp, 2023; Grant & Spivey, 2003). Problem-based learning anchored in authentic community needs operationalizes this paradox: when students design interventions within economic, social, and ethical constraints, they develop attunement to feasible creativity where usefulness provides grounding enabling sustained novelty exploration, while novelty infuses usefulness with transformative potential. Rather than viewing constraints as creativity inhibitors, educators must recognize their dynamic interdependence as the source of adaptive originality.

The persistence–flexibility paradox necessitates educational programs embedding longitudinal, iterative projects that immerse students in recursive ideation–refinement cycles across extended timeframes. Structuring endeavors around reflective checkpoints, peer critique sessions, and formal revision opportunities reframes failure as a productive developmental phase, enabling students to develop metacognitive awareness for monitoring cognitive states and strategically shifting between exploratory and exploitative modes (Glăveanu, 2010; Nijstad et al., 2010). This dual engagement proves especially critical as task complexity increases: simple tasks may succeed through linear approaches emphasizing either flexibility or persistence, but complex activities demand dynamic oscillation between both poles (Steele et al., 2021). Educators must cultivate the capacity to discern when to intensify effort and when to recalibrate direction, recognizing that optimal creativity emerges not from favoring one pole but from fluid alternation responsive to evolving task demands. Research reveals emphasizing competence development over performance demonstration could more effectively promote exploratory and exploitative effort because both contribute to learning (Steele et al., 2021), implying that educational contexts prioritizing deep understanding over ability comparison enable students to sustain the productive tension between persistence and flexibility.

The diversity–resonance paradox requires collaborative learning structures that transcend conventional group work toward intentional co-creation fostering psychological safety, shared mindset, and coordinated engagement. Teams adopting paradoxical frames—mental templates recognizing and embracing contradictions—combined with high epistemic motivation develop more creative solutions by actively exchanging, considering, and integrating diverse ideas rather than settling for superficial compromises (Miron-Spektor et al., 2022). Educational practices including rotating leadership, flipped teaching, dialogic protocols, and collaborative improvisation synchronize heterogeneous perspectives without homogenizing them, teaching students to navigate creative tensions and reframe difference as generative resource rather than obstacle. Neuroscientific evidence demonstrates that optimal divergence—neither minimal nor maximal—promotes alignment of neural and creative processes: moderately diverse teams achieve highest inter-brain synchrony and creative outcomes, whereas extremely homogeneous or diverse teams prove less effective (Lu et al., 2021, 2023). This finding suggests that educators must strategically compose and scaffold collaborative configurations, recognizing that diversity alone is insufficient—teams must cultivate resonance through shared goals, transparent communication, and practices amplifying emotional attunement and coordinated problem-solving. By integrating these paradox-informed principles, educators equip learners to sustain innovation, adapt flexibly, and collaboratively transform knowledge into action—embracing rather than resolving the fundamental tensions that constitute creativity.

## Figures and Tables

**Table 1:** Major Publication Sources on the Paradox of Creativity.

Rank	Publication Sources	n
1	Creativity and Innovation Management	19
2	Frontiers in Psychology	12
3	Journal of Creative Behavior	11
4	Creativity Research Journal	9
5	Organizational Behavior and Human Decision Processes	9
6	Psychology of Aesthetics Creativity and the Arts	7
7	Journal of Organizational Behavior	6
8	Human Relations	5
9	Journal of Product Innovation Management	4
10	Journal of Applied Behavioral Science	4
11	Management Decision	4
12	Organization Studies	4
13	Current Psychology	4
14	Journal of Organizational Change Management	3
15	IEEE Transactions on Engineering Management	3
16	Industry and Innovation	3
17	International Journal of Arts Management	3
18	Neuroscience and Biobehavioral Reviews	3
19	Thinking Skills and Creativity	3
20	Academy of Management Journal	3
21	Academy of Management Review	3

**Table 2:** Disciplinary Distribution of Research on the Paradox of Creativity.

Rank	Research Area	n
1	Business & Economics	101
2	Psychology	58
3	Psychology; Business & Economics	13
4	Business & Economics; Social Sciences—Other Topics	10
5	Education & Educational Research	10
6	Business & Economics; Engineering	8
7	Business & Economics; Psychology	7
8	Arts & Humanities—Other Topics; Psychology	7
9	Social Sciences—Other Topics	6
10	Psychiatry	6
11	Neurosciences & Neurology	5
12	Cultural Studies	4
13	Computer Science; Information Science & Library Science	4
14	Behavioral Sciences; Psychology; Business & Economics	4
15	Information Science & Library Science; Business & Economics	3
16	Engineering; Business & Economics; Operations Research & Management Science	3
17	Business & Economics; Science & Technology—Other Topics	3
18	Business & Economics; Development Studies	3
19	Business & Economics; Communication	3
20	Behavioral Sciences; Neurosciences & Neurology	3
21	Arts & Humanities—Other Topics; Business & Economics	3

**Table 3:** Representative Studies Addressing Different Paradoxes of Creativity.

No.	Topic	Study	Key Findings
1	Paradox of creativity	Bonetto et al. (2021)	Creativity is an evolutionary paradox: it enhances survival and reproduction but incurs social and adaptive costs, persisting as a trait balanced between benefits and risks.
2	Paradox of creativity	Brillenburg Wurth (2019)	Creativity emerges from irresolvable tensions and Janusian thinking, with paradox bridging artistic and scientific domains and shaping creativity as a situated, multidimensional, reality-testing process.
3	Paradox of creativity	Barzun (1989)	Creativity is inherently paradoxical—disciplined yet spontaneous, individual yet collective, rule-bound yet rule-breaking. True creativity arises from balancing freedom with constraint and innovation with tradition, with these contradictions acting as essential drivers of creative processes and cultural evolution.
4	Novelty vs. usefulness	Fei et al. (2025)	Creativity reflects a novelty–utility paradox: their alignment enhances flow, improving in-role performance, while learning goal orientation moderates this effect, weakening the flow–performance link for highly learning-oriented individuals.
5	Novelty vs. usefulness	Geng et al. (2025)	Geng et al. (2025) find team radical creativity relies on balancing originality and usefulness, with high error management and moderate error aversion promoting information exchange to resolve this paradox.
6	Novelty vs. usefulness	Steele et al. (2021)	Steele et al. (2021) show that managing learning–performance, exploration–exploitation, and novelty–usefulness paradoxes can substantially boost creativity, with mastery goals and self-regulation balancing efforts.
7	Diversity vs. resonance	Mannucci and Shalley (2022)	Teams with high multicultural paradox mindsets enhance creativity by embracing intercultural tensions and fostering information elaboration, allowing diverse teams to transform conflicts into productive outcomes.
8	Diversity vs. resonance	Miron-Spektor et al. (2022)	Miron-Spektor et al. (2022) found that teams with paradoxical frames and high epistemic motivation boost creativity by integrating diverse perspectives, while lacking either factor leads to suboptimal solutions.
9	Diversity vs. resonance	Gomez Celis et al. (2025)	The paradoxical frames boost team creativity by fostering cognitive conflict and integrative complexity, enabling deep engagement with diverse perspectives and synthesis of opposing viewpoints.
10	Flexibility vs. persistence	Calic et al. (2019)	The study finds that paradoxical frames influence creativity nonlinearly, enhancing idea generation via differentiation and integration but potentially suppressing it, revealing a conditional link between paradoxical thinking and creative performance.
11	Flexibility vs. persistence	Miron-Spektor (2025)	The chapter shows that paradoxical frames, by integrating contradictions, boost creativity across innovation phases, with cultural and contextual factors shaping their effectiveness.
12	Flexibility vs. persistence	Guo et al. (2025)	The study shows that paradoxical tensions boost engineering students’ creativity via paradoxical thinking, moderated by team psychological capital, revealing key cognitive mechanism and informing educational strategies.

## Data Availability

The data presented in this study are available on request from the corresponding author.

## References

[B1-behavsci-16-00129] Agnati L. F., Barlow P., Ghidoni R., Borroto-Escuela D. O., Guidolin D., Fuxe K. (2012). Possible genetic and epigenetic links between human inner speech, schizophrenia and altruism. Brain Research.

[B2-behavsci-16-00129] Amabile T. M., Conti R., Coon H., Lazenby J., Herron M. (1996). Assessing the work environment for creativity. Academy of Management Journal.

[B3-behavsci-16-00129] Andriopoulos C., Lewis M. W. (2010). Managing innovation paradoxes: Ambidexterity lessons from leading product design companies. Long Range Planning.

[B4-behavsci-16-00129] Barzun J. (1989). The paradoxes of creativity. The American Scholar.

[B5-behavsci-16-00129] Baumeister R. F., Leary M. R. (1997). Writing narrative literature reviews. Review of General Psychology.

[B6-behavsci-16-00129] Beaty R. E., Benedek M., Barry Kaufman S., Silvia P. J. (2015). Default and executive network coupling supports creative idea production. Scientific Reports.

[B7-behavsci-16-00129] Beaty R. E., Benedek M., Silvia P. J., Schacter D. L. (2016). Creative cognition and brain network dynamics. Trends in Cognitive Sciences.

[B8-behavsci-16-00129] Beaty R. E., Seli P., Schacter D. L. (2019). Network neuroscience of creative cognition: Mapping cognitive mechanisms and individual differences in the creative brain. Current Opinion in Behavioral Sciences.

[B9-behavsci-16-00129] Bonetto E., Pichot N., Pavani J. B., Adam-Troïan J. (2021). The paradox of creativity. New Ideas in Psychology.

[B10-behavsci-16-00129] Brillenburg Wurth K. (2019). The creativity paradox: An introductory essay. Journal of Creative Behavior.

[B11-behavsci-16-00129] Brüne M. (2004). Schizophrenia—An evolutionary enigma?. Neuroscience & Biobehavioral Reviews.

[B12-behavsci-16-00129] Calic G., Hélie S. (2018). Creative sparks or paralysis traps? The effects of contradictions on creative processing and creative products. Frontiers in Psychology.

[B13-behavsci-16-00129] Calic G., Hélie S., Bontis N., Mosakowski E. (2019). Creativity from paradoxical experience: A theory of how individuals achieve creativity while adopting paradoxical frames. Journal of Knowledge Management.

[B14-behavsci-16-00129] Calic G., Shevchenko A., Ghasemaghaei M., Bontis N., Ozmen Tokcan Z. (2020). From sustainability constraints to innovation: Enhancing innovation by simultaneously attending to sustainability and commercial imperatives. Sustainability Accounting, Management and Policy Journal.

[B15-behavsci-16-00129] Dietrich A., Kanso R. (2010). A review of EEG, ERP, and neuroimaging studies of creativity and insight. Psychological Bulletin.

[B16-behavsci-16-00129] Donthu N., Kumar S., Mukherjee D., Pandey N., Lim W. M. (2021). How to conduct a bibliometric analysis: An overview and guidelines. Journal of Business Research.

[B17-behavsci-16-00129] Duan C., Zhang M. J., Liu X., Ling C. D., Xie X. Y. (2023). Investigating the curvilinear relationship between temporal leadership and team creativity: The moderation of knowledge complexity and the mediation of team creative process engagement. Journal of Organizational Behavior.

[B18-behavsci-16-00129] Fei X., Wang J., Zhu Y., Chen T. (2025). Examining creativity as a paradox: The interactive effects of novelty and utility on in-role performance. Journal of Creative Behavior.

[B19-behavsci-16-00129] Fink A., Benedek M. (2014). EEG alpha power and creative ideation. Neuroscience & Biobehavioral Reviews.

[B20-behavsci-16-00129] Galang A. J. R. (2010). The prosocial psychopath: Explaining the paradoxes of the creative personality. Neuroscience & Biobehavioral Reviews.

[B21-behavsci-16-00129] Geng Z., Xiao M., Wang H., Xue J., Wang Y. (2025). Navigating the paradox of originality and usefulness: The roles of distinct error climates in fostering team radical creativity. Creativity and innovation management.

[B22-behavsci-16-00129] Glăveanu V. P. (2010). Paradigms in the study of creativity: Introducing the perspective of cultural psychology. New Ideas in Psychology.

[B23-behavsci-16-00129] Gomez Celis D. A., Liou S., Hernandez Sibo I. P. (2025). Enhancing team creativity through paradoxical frames: Exploring the role of conflict and integrative complexity. Journal of Creative Behavior.

[B24-behavsci-16-00129] Grant E. R., Spivey M. J. (2003). Eye movements and problem solving: Guiding attention guides thought. Psychological Science.

[B25-behavsci-16-00129] Guo H., Zhou Z., Ma F. (2025). Exploring the roles of paradoxical tensions, paradoxical thinking, and team psychological capital on the creativity of engineering university students. BMC Psychology.

[B26-behavsci-16-00129] Guyon H., Kop J. L., Juhel J., Falissard B. (2018). Measurement, ontology, and epistemology: Psychology needs pragmatism-realism. Theory & Psychology.

[B27-behavsci-16-00129] Harvey S., Berry J. W. (2023). Toward a meta-theory of creativity forms: How novelty and usefulness shape creativity. Academy of Management Review.

[B28-behavsci-16-00129] Heradio R., De La Torre L., Galan D., Cabrerizo F. J., Herrera-Viedma E., Dormido S. (2016). Virtual and remote labs in education: A bibliometric analysis. Computers & Education.

[B29-behavsci-16-00129] Huang F., Fan J., Luo J. (2015). The neural basis of novelty and appropriateness in processing of creative chunk decomposition. Neuroimage.

[B30-behavsci-16-00129] Huang F., Tang S., Sun P., Luo J. (2018). Neural correlates of novelty and appropriateness processing in externally induced constraint relaxation. NeuroImage.

[B31-behavsci-16-00129] Jehn K. A., Mannix E. A. (2001). The dynamic nature of conflict: A longitudinal study of intragroup conflict and group performance. Academy of Management Journal.

[B32-behavsci-16-00129] Joshi A., Roh H. (2009). The role of context in work team diversity research: A meta-analytic review. Academy of Management Journal.

[B33-behavsci-16-00129] Kamil N. L. M., Zhao K., Nordin W. N. A. W. M., Idris M. A. (2025). Leading with paradox: Promoting self-leadership and positive work behaviours through leader-member exchange. Journal of Occupational and Organizational Psychology.

[B34-behavsci-16-00129] Kearney E., Shemla M., van Knippenberg D., Scholz F. A. (2019). A paradox perspective on the interactive effects of visionary and empowering leadership. Organizational Behavior and Human Decision Processes.

[B35-behavsci-16-00129] Knoblich G., Haider H. (2019). Empirical evidence for constraint relaxation in insight problem solving. Proceedings of the eighteenth annual conference of the cognitive science society.

[B36-behavsci-16-00129] Lambert P. A. (2020). The order-chaos dynamic of creativity. Creativity Research Journal.

[B37-behavsci-16-00129] Leung A. K.-y., Liou S., Miron-Spektor E., Koh B., Chan D., Eisenberg R., Schneider I. (2018). Middle ground approach to paradox: Within- and between-culture examination of the creative benefits of paradoxical frames. Journal of Personality and Social Psychology.

[B38-behavsci-16-00129] Liu Y., Xu S., Zhang B. (2020). Thriving at work: How a paradox mindset influences innovative work behavior. The Journal of Applied Behavioral Science.

[B39-behavsci-16-00129] Lu K., Gao Z., Wang X., Qiao X., He Y., Zhang Y., Hao N. (2023). The hyper-brain neural couplings distinguishing high-creative group dynamics: An fNIRS hyperscanning study. Cerebral Cortex.

[B40-behavsci-16-00129] Lu K., Qiao X., Yun Q., Hao N. (2021). Educational diversity and group creativity: Evidence from fNIRS hyperscanning. NeuroImage.

[B41-behavsci-16-00129] Ma H., Ismail L. (2025). Bibliometric analysis and systematic review of digital competence in education. Humanities and Social Sciences Communications.

[B42-behavsci-16-00129] Manca C. (2022). Tensions as a framework for managing work in collaborative workplaces: A review of the empirical studies. International Journal of Management Reviews.

[B43-behavsci-16-00129] Mannucci P. V., Shalley C. E. (2022). Embracing multicultural tensions: How team members’ multicultural paradox mindsets foster team information elaboration and creativity. Organizational Behavior and Human Decision Processes.

[B44-behavsci-16-00129] Michinov N. (2012). Is electronic brainstorming or brainwriting the best way to improve creative performance in groups? An overlooked comparison of two idea-generation techniques. Journal of Applied Social Psychology.

[B45-behavsci-16-00129] Miron-Spektor E., Gelfand M. J., Chiu C.-Y., Hong Y. (2025). Managing paradoxes of creativity and innovation. Handbook of advances in culture and psychology.

[B46-behavsci-16-00129] Miron-Spektor E., Emich K. J., Argote L., Smith W. K. (2022). Conceiving opposites together: Cultivating paradoxical frames and epistemic motivation fosters team creativity. Organizational Behavior and Human Decision Processes.

[B47-behavsci-16-00129] Miron-Spektor E., Ingram A., Keller J., Smith W. K., Lewis M. W. (2018). Microfoundations of organizational paradox: The problem is how we think about the problem. Academy of Management Journal.

[B48-behavsci-16-00129] Mueller J. S., Melwani S., Goncalo J. A. (2012). The bias against creativity: Why people desire but reject creative ideas. Psychological Science.

[B49-behavsci-16-00129] Nijstad B. A., De Dreu C. K., Rietzschel E. F., Baas M. (2010). The dual pathway to creativity model: Creative ideation as a function of flexibility and persistence. European Review of Social Psychology.

[B50-behavsci-16-00129] O’Reilly C. A., Williams K. Y., Barsade S., Gruenfeld D. (1998). Group demography and innovation: Does diversity help?. Composition.

[B51-behavsci-16-00129] Osorio A., Bornmann L. (2021). On the disruptive power of small-teams research. Scientometrics.

[B52-behavsci-16-00129] Potonik K., Verwaeren B., Nijstad B. (2022). Tensions and paradoxes in creativity and innovation. Journal of Work and Organizational Psychology.

[B53-behavsci-16-00129] Reif J. A., Feldmeier C. L., Georganta E., Kugler K. G., Brodbeck F. C. (2025). From ideation to implementation: A model of team innovation. Group & Organization Management.

[B54-behavsci-16-00129] Ren J., Huang F., Zhou Y., Zhuang L., Xu J., Gao C., Qin S., Luo J. (2020). The function of the hippocampus and middle temporal gyrus in forming new associations and concepts during the processing of novelty and usefulness features in creative designs. NeuroImage.

[B55-behavsci-16-00129] Revilla E., Rodriguez-Prado B. (2018). Bulding ambidexterity through creativity mechanisms: Contextual drivers of innovation success. Research Policy.

[B56-behavsci-16-00129] Rothenberg A. (1996). The Janusian process in scientific creativity. Creativity Research Journal.

[B57-behavsci-16-00129] Runco M., Jaeger G. (2012). The standard definition of creativity. Creativity Research Journal.

[B58-behavsci-16-00129] Schoug A., Avby G., Rubin V., Ohlsson J. (2025). Collective creativity through a learning lens: Paradoxes of convergence and divergence in an art and theater project. Management Learning.

[B59-behavsci-16-00129] Shen W., Yuan Y. (2015). Sociocultural basis underlying creative thinking. Advances in Psychological Science.

[B60-behavsci-16-00129] Shen W., Yuan Y., Liu C., Luo J. (2016). In search of the “Aha!” experience: Elucidating the emotionality of insight problem-solving. British Journal of Psychology.

[B61-behavsci-16-00129] Shen W., Yuan Y., Liu C., Luo J. (2017). The roles of the temporal lobe in creative insight: An integrated review. Thinking & Reasoning.

[B62-behavsci-16-00129] Shen W., Yuan Y., Yi B., Liu C., Zhan H. (2019). A theoretical and critical examination on the relationship between creativity and morality. Current Psychology.

[B63-behavsci-16-00129] Simonton D. K. (2012). Taking the US Patent Office criteria seriously: A quantitative three-criterion creativity definition and its implications. Creativity Research Journal.

[B64-behavsci-16-00129] Simonton D. K. (2014). The mad-genius paradox: Can creative people be more mentally healthy but highly creative people more mentally ill?. Perspectives on Psychological Science.

[B65-behavsci-16-00129] Smith W. K., Lewis M. W. (2011). Toward a theory of paradox: A dynamic equilibrium model of organizing. Academy of Management Review.

[B66-behavsci-16-00129] Steele L. M., Hardy J. H., Day E. A., Watts L. L., Mumford M. D. (2021). Navigating creative paradoxes: Exploration and exploitation effort drive novelty and usefulness. Psychology of Aesthetics, Creativity, and the Arts.

[B67-behavsci-16-00129] Thys E., Sabbe B., De Hert M. (2013). Creativity and psychiatric illness: The search for a missing link-an historical context for current research. Psychopathology.

[B68-behavsci-16-00129] Tromp C. (2023). Integrated constraints in creativity: Foundations for a unifying model. Review of General Psychology.

[B69-behavsci-16-00129] Volpentesta T., Spahiu E., De Giovanni P. (2023). A survey on incumbent digital transformation: A paradoxical perspective and research agenda. European Journal of Innovation Management.

[B70-behavsci-16-00129] Wang J., Kim T. Y., Bateman T. S., Jiang Y., Tang G. (2024). A paradox theory lens on proactivity, individual ambidexterity, and creativity: An empirical look. Journal of Organizational Behavior.

[B71-behavsci-16-00129] Weisberg R. W. (2015). On the usefulness of “value” in the definition of creativity. Creativity Research Journal.

[B72-behavsci-16-00129] West M. A., Richter A. W. (2011). Team climate and effectiveness outcomes. Handbook of organizational culture and climate.

[B73-behavsci-16-00129] Westby E. L., Dawson V. L. (1995). Creativity: Asset or burden in the classroom?. Creativity Research Journal.

[B74-behavsci-16-00129] Wiggins G. A., Bhattacharya J. (2014). Mind the gap: An attempt to bridge computational and neuroscientific approaches to study creativity. Frontiers in Human Neuroscience.

[B75-behavsci-16-00129] Wu Y., Koutstaal W. (2022). Creative flexibility and creative persistence: Evaluating the effects of instructed vs autonomous choices to shift vs. dwell on divergent and convergent thinking. Consciousness and Cognition.

[B76-behavsci-16-00129] Zabelina D. L., Robinson M. D. (2010). Creativity as flexible cognitive control. Psychology of Aesthetics, Creativity, and the Arts.

[B77-behavsci-16-00129] Zhang C., Shao Y., Yuan Y., Shen W. (2025). Artificial intelligence reshapes creativity: A multidimensional evaluation. PsyCh Journal.

[B78-behavsci-16-00129] Zhang W., Sjoerds Z., Hommel B. (2020). Metacontrol of human creativity: The neurocognitive mechanisms of convergent and divergent thinking. NeuroImage.

[B79-behavsci-16-00129] Zhang Y., Ye W., Yin J., Wu Q., Huang Y., Hao N., Cui L., Zhang M., Cai D. (2024). Exploring the role of mutual prediction in inter-brain synchronization during competitive interactions: An fNIRS hyperscanning investigation. Cerebral Cortex.

[B80-behavsci-16-00129] Zhao R., Tang Z., Lu F., Xing Q., Shen W. (2022). An updated evaluation of the dichotomous link between creativity and mental health. Frontiers in Psychiatry.

